# Intervention accelerator to prevent and respond to abuse of older people: insights from key promising interventions

**DOI:** 10.1016/j.lanhl.2024.100647

**Published:** 2024-12-13

**Authors:** Laura Campo-Tena, Aresya Farzana, David Burnes, Titus A Chan, Wan Yuen Choo, Mélanie Couture, Fatemeh Estebsari, Minying He, Jeffrey H Herbst, Christelle Sibdou Liliane Kafando, Joshua Lachs, George Rouamba, Marie-Madeleine Simbreni, Louis To, Hau Yan Wan, Elsie Yan, Yongjie Yon, Christopher Mikton

**Affiliations:** Institute of Criminology, University of Cambridge, Cambridge, UK (L Campo-Tena PhD); Department of Social and Preventive Medicine, Universiti Malaya, Kuala Lumpur, Malaysia (A Farzana BSc, Prof W Y Choo PhD); Factor-Inwentash Faculty of Social Work, University of Toronto, Toronto, ON, Canada (Prof D Burnes PhD, T A Chan MSW); École de travail social, Université de Sherbrooke, Sherbrooke, QC, Canada (Prof M Couture PhD); School of Nursing and Midwifery, Medical Ethics and Law Research Center, Shahid Beheshti University of Medical Sciences, Tehran, Iran (F Estebsari PhD); Centers for Disease Control and Prevention, Atlanta, GA, USA (J H Herbst PhD); Institut Supérieur des Sciences de la Population, Université Joseph Ki-Zerbo, Ouagadougou, Burkina Faso (C S L Kafando MA); Weill Cornell Medicine, New York, NY, USA (J Lachs BA); Department of Sociology, Université Joseph Ki-Zerbo, Ouagadougou, Burkina Faso (G Rouamba PhD, M M Simbreni BA); Department of Applied Social Sciences, Hong Kong Polytechnic University, Hong Kong Special Administrative Region, China (M He MSc, L To BSW, H Y Wan MSc, Prof E Yan PhD); Division of Country Health Policies and Systems, WHO Regional Office for Europe, Copenhagen, Denmark (Y Yon PhD); Department of Social Determinants of Health, Division of Healthier Populations, WHO, Geneva, Switzerland (C Mikton PhD)

## Abstract

Globally, abuse of older people (AOP) affects one in six individuals aged 60 years and older every year. Despite the widespread prevalence of AOP, evidence-based interventions for preventing and responding to this issue are insufficient. To address this gap, WHO proposed an initiative to accelerate the development of effective interventions for AOP across all country income levels. In the first phase, the initiative identified 89 promising interventions across a total of 101 evaluations or descriptions, which led to the creation of a public database. Most interventions targeted physical, psychological, and financial abuse and neglect, were implemented in the USA, and focused on victims or potential victims. These interventions were primarily delivered by social workers and nurses, usually in health-care facilities and community centres. Face-to-face delivery was common. Additionally, 28 (28%) of the 101 evaluations used randomised controlled trial designs. The results of this Review can be used to identify interventions that are ready for a rigorous outcome evaluation.

## Introduction

The scarcity of evidence-based interventions for preventing and responding to the abuse of older people (AOP) is a pressing issue. Older people in this context include individuals aged 60 years and older, in line with the UN definition of older people. Systematic reviews have consistently highlighted the absence of effective interventions assessed in high-quality evaluations.^1–7^ In 2022, WHO initiated a priority setting exercise and convened a 50-member group of experts and stakeholders from across the world, including academics, policy makers, and funders, who unanimously recognised the development of effective interventions as a top priority for preventing AOP globally.^8^

The absence of effective and cost-effective interventions could lead to deprioritisation of AOP initiatives by governments, international and civil society organisations, and donors. Acknowledging this concern, WHO launched a global initiative, within the UN Decade of Healthy Ageing (2021–30), to establish an intervention accelerator to prevent abuse of older people (AOP-IA). The intervention accelerator aims to speed up the development of effective interventions to prevent and reduce AOP in various settings across countries of different income levels.

The AOP-IA project has four distinct phases: identification and creation of a database of promising intervention approaches (ie, interventions that can achieve relevant outcomes and that meet the inclusion criteria decided upon) and the selection of a subset of candidate interventions for further refinement, adaptation, and rigorous testing; creation and maintenance of a network of developers, implementers, and evaluators of intervention approaches; refinement and adaptation of candidate intervention approaches and their evaluation through rigorous pilot testing; and creation and maintenance of an online portfolio of interventions that will be updated regularly.^8^ This Review aims to describe promising interventions included in the database created in phase 1 of the intervention accelerator.

## Methods

### Search strategy and selection criteria

A systematic four-step strategy was adopted to identify interventions that address the prevention of AOP.

First, the primary studies included in published systematic reviews of interventions (n=53) were identified on the basis of a mega-map that covers the prevalence, consequences, and risk and protective factors of AOP, as well as relevant interventions. The search strategy is described in detail in the published protocol.^9^

Second, updated searches were conducted for primary studies on interventions that were published between Jan 1, 2013, and June 1, 2023. Although the searches were conducted in English, language restrictions were not included in our eligibility criteria. The databases that were searched included Academic Search Premier, Cumulative Index in Nursing and Allied Health Literature, Cochrane Library, Embase, MEDLINE, PsycINFO, PubMed, Scopus, Sociological Abstracts, and Web of Science. The searches were adapted for each database and included a combination of terms referring to AOP, types of abuse, and the relevant vocabulary on the topic of interventions (eg, crisis intervention, primary intervention).

Third, WHO issued an international call to experts in the field to help to identify additional studies of interventions for preventing AOP. Experts identified through established AOP listservs and networks were provided with detailed project information, objectives, and a link to an online form for submitting their suggestions for possible interventions, which was connected to the AirTable database for this study.

Finally, we also searched, screened, and extracted data on interventions from the Violence Prevention Information System (Violence Info), a global knowledge platform that compiles scientific studies on prevention of interpersonal violence (including prevention of AOP).^10^

The project team developed selection criteria for identifying promising interventions and shared them with a 12-member group of experts for review. These experts are regular collaborators on the AOP-IA project and specialise in addressingAOP in diverse regions of the world, including North America, Europe, Africa, and Asia.

The inclusion criteria were interventions for preventing or responding to AOP (aged ≥ 60 years); interventions that targeted older individuals at risk for facing abuse (ascertained by differentiating primary prevention interventions that targeted older adults who had not been victimised from secondary or tertiary prevention interventions that targeted victims of abuse), caregivers, relatives, and institutions; and interventions that were evaluated with a quantitative research design for causal inference and that showed a substantial positive effect or interventions based on a detailed logic model or programme theory (ie, visual representation or assumptions outlining how the intervention functions and is expected to achieve its goals) with some empirical support or previous positive evaluation (appendix pp 2–8). These interventions based on logical models or programme theories were included because they had been designed following best practices and evidence-based models that suggested a high likelihood of effectiveness, even in the absence of direct outcome measurements in some publications. In some instances, the interventions were grounded in research or theoretical frameworks that had shown success in related contexts. Two independent researchers (CM and LC-T) then piloted the selection criteria on a subset of 20 studies.

Exclusion criteria ruled out reviews and protocols; interventions on populations aged younger than 60 years (without age-disaggregated findings); interventions in which there was no expectation of trust between the victim and the perpetrators involved, such as a street crime perpetrated by strangers, as this type of scenario does not align with the definition of AOP; interventions that addressed self-neglect, ageism, or use of restraint or seclusion; studies that evaluated instruments to detect AOP; and studies with statistically insignificant effects or negative effects, those with qualitative designs, or those that did not have a well-described logic model. An expectation of trust was defined as a relationship in which the victim has a reasonable belief in the reliability, honesty, or integrity of the perpetrator. The perpetrator could include family members, friends, professional or non-professional caregivers, or individuals in positions of authority or perceived authority. Financial or material abuse, including scams and frauds, were not excluded as the older person has an expectation of trust (although misplaced) with the person who the scammer or fraudster was purporting to be.

Guided by the detailed eligibility criteria, the project team performed a screening of 13 940 records obtained through the specified four steps, which was conducted in duplicate using Microsoft Excel. Two additional team members who had contributed to the development of the screening criteria (CM and LC-T) were present at these meetings and engaged in discussions to facilitate consensus, when necessary.

### Data extraction

The project team developed a coding scheme(appendix pp 9–15) for extracting details from the interventions selected, which was piloted on a subset before implementation. The data extracted included the name of the intervention, key personnel, adaptation status, intervention aim, targeted abuse type, beneficiary age, intervention delivery agents, setting, training requirements, theoretical basis, materials used, procedures, mode of delivery, location details (ie, WHO region, World Bank income level, country, city or subnational entity, and urban or rural), language of evaluation, delivery specifics (ie, timing, session count, schedule, and duration), details of the process evaluation, and details of the outcome evaluation. To verify the quality of the dataset, assessments were performed on 20% of the data by a second researcher. A third researcher (CM or LC-T) was involved in the discussion of any incongruencies.

### Data management and analysis

AirTable, a cloud-based platform, was used to organise, structure, and manage the extracted data. The database of promising interventions is publicly accessible, with information sourced from the WHO website. The characteristics of the interventions identified through the search and selection process were analysed using descriptive statistics in Microsoft Excel.

## Results

### Study selection and characteristics

**Figure 1:**
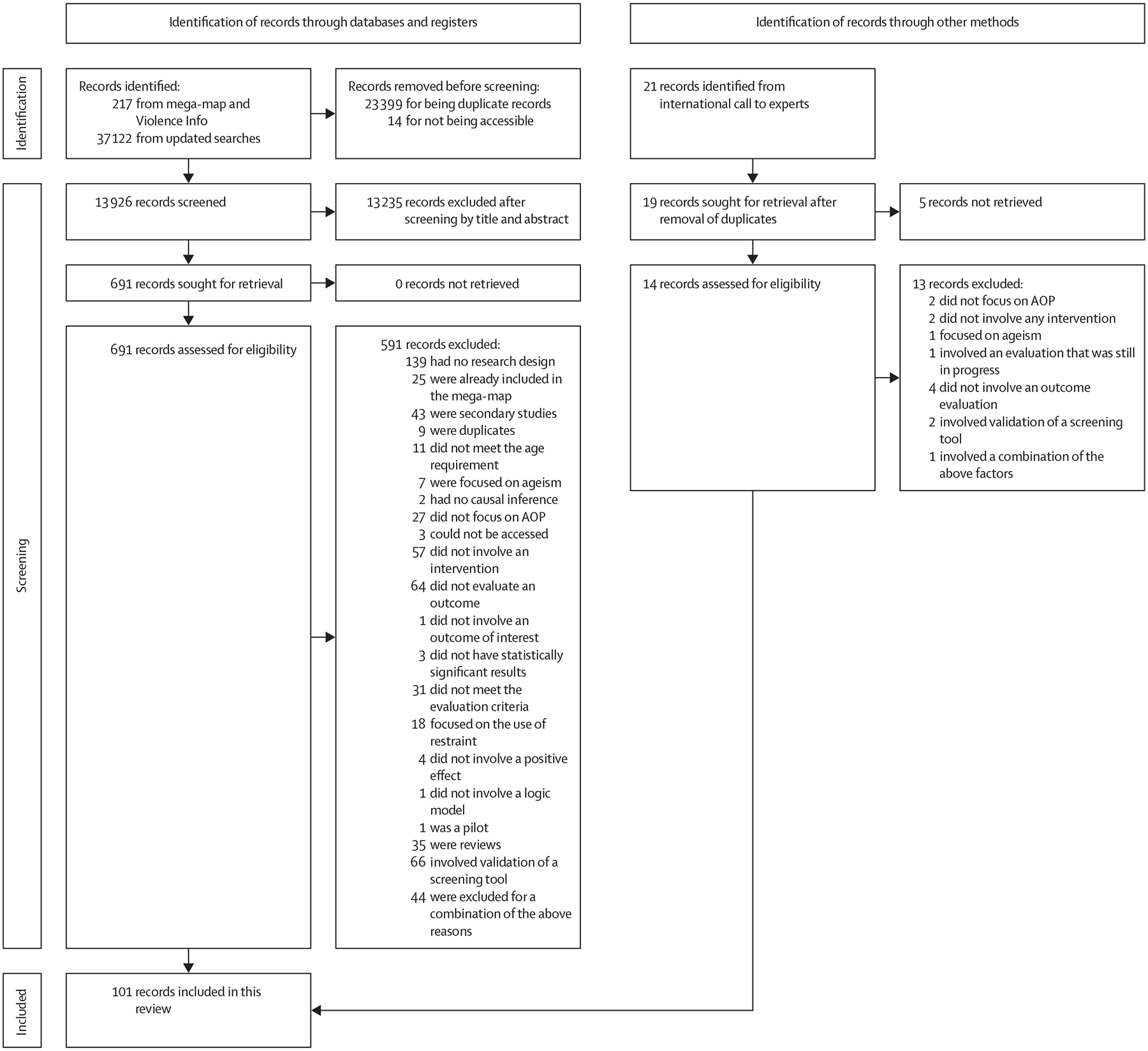
Diagram of the process for the selection of promising interventions The intervention evaluations or descriptions included in this Review were identified through mega-map and Violence Info, updated searches, and an international call to experts. The term “records” refers to any published evaluations or descriptions of interventions to prevent or address AOP. AOP=abuse of older people.

After removing duplicates, 13 945 titles and abstracts were screened for eligibility (figure 1): 191 from the mega-map, 13 735 from the updated searches, and 19 from the international call to experts initiated by WHO. On applying the selection criteria to the initial search results, 710 primary studies advanced to the next screening stage and underwent full-text screening. Of these, 101 evaluations or descriptions of interventions met the selection criteria and were included (appendix pp 16–26). A substantial level of agreement was found between reviewers (k = 0·77); any disagreements were resolved through discussion or by involving a third researcher (CM or LC-T), when necessary.

Most evaluations or descriptions of interventions included in our analysis originated from the mega-map of systematic reviews (n=52) and updated searches (n=48), and only one was incorporated from the international call to experts. As all interventions listed on the Violence Info platform were already identified through the mega-map, no additional studies were selected from the platform.

In total, the search process identified 89 unique promising interventions reported in 99 different publications. Of these, one publication^11^ contained three different evaluations or descriptions of interventions, resulting in a total of 101 evaluations or descriptions. These studies were published between 1993 and 2023. Between 1993 and 2009, the publication rate remained low and stable. However, a noticeable increase was seen in the number of evaluations or descriptions published on interventions for preventing and responding to AOP between 2010 and 2019, reaching a peak in 2015–19 (appendix p 27).

Nearly half (50 [50%]) of the 101 included evaluations or descriptions reported secondary prevention interventions focused on immediate responses to AOP. Primary prevention interventions (those aiming to prevent abuse before it occurs) were reported in 30 (30%) of 101 evaluations or descriptions; tertiary prevention interventions (those focused on long-term care following abuse) were reported in 21 (21%) of 101 evaluations or descriptions.

This Review aimed to include research from diverse regions; however, we found that most (61 [60%] of 101) evaluations or descriptions of interventions that met our inclusion criteria were conducted in the region of the Americas, mainly in the USA (53 [53%]; appendix p 28). 13 (13%) of 101 evaluations were done in the European region. An equal number of interventions were from the Western Pacific and Eastern Mediterranean regions (11 [11%] for both), with many from Iran (ten [10%]). Five (5%) of 101 evaluations were from the South-East Asia region, primarily from India (two [2%]) and Indonesia (three [3%]). No interventions were found from the African region.

Based on the 2022 World Bank income classification, most evaluations or descriptions were conducted in high-income countries (HICs; 80 [79%] of 101), with the remaining done in lower-middle-income countries (13 [13%] of 101) and upper-middle-income countries (eight [8%] of 101). No evaluations were conducted in low-income countries. Although most of the evaluations or descriptions of interventions (97 [96%] of 101) were published in English, two were published in Spanish,^12,13^ one in Norwegian,^14^ and one in Persian.^15^

### Interventions

35 (35%) of the 101 intervention evaluations or descriptions referred to original interventions—ie, the intervention was delivered in the original form with no adaptations, and 29 (29%) considered adaptations of existing interventions. The remaining 37 (37%) did not explicitly report how the intervention originated.

**Figure 2:**
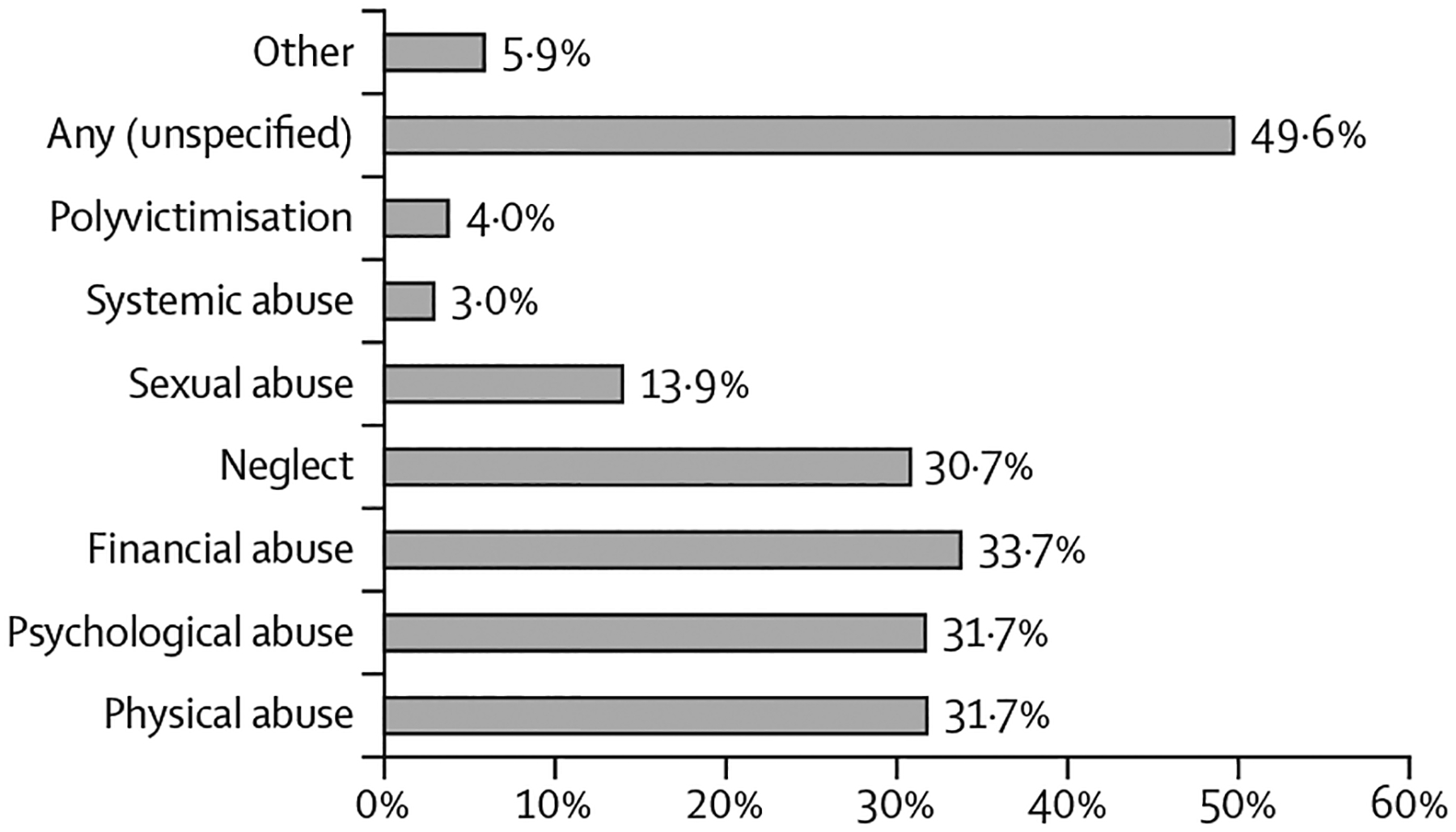
Types of abuse targeted in the promising interventions The results are given as a percentage of the 101 intervention evaluations or descriptions identified in this Review.

With respect to the types of abuse addressed (figure 2), nearly half (50 [50%] of the 101 evaluations or descriptions) used interventions that targeted any form of abuse, in some cases without specifying the types of abuse in question. When specific types of abuse were reported, physical abuse (32 [32%] of 101), psychological abuse (32 [32%]), financial abuse (34 [34%]), and neglect (31 [31%]) were the most common. In contrast, interventions that addressed sexual abuse accounted for 14 (14%), and those addressing systemic, organisational, and institutional abuse constituted the smallest proportion (three [3%]). Polyvictimisation (4 [4%]) included multiple types of abuse. The category named “other” (6 [6%]) included definitions other than those specified previously, such as violation of rights, social problems, and online assaults.

#### Victims and perpetrators of abuse involved

17 (17%) of the 101 intervention evaluations or descriptions of the promising interventions analysed targeted older victims of abuse and 31 (31%) targeted older people at risk of becoming victims. Over half of the evaluations of interventions (52 [52%] of 101) predominantly or exclusively involved women; half of these interventions (26 [50%] of 52) targeted older people at risk of being abused. Other participants included the general population (ten [10%] of 101), non-professional caregivers at risk of perpetrating abuse (nine [9%]), professional caregivers at risk of perpetrating abuse (18 [18%]), institutions (ten [10%]), and perpetrators of abuse (three [3%]). 16 (16%) focused on concerned individuals, including those who were involved in a situation of abuse directed at an older person but did not perpetrate the abuse, such as friends, neighbours, and relatives of the older victim of abuse.

Intervention deliverers included primarily social workers (18 [18%] of 101), followed by educators (14 [14%]), nurses (13 [13%]), and other health-care workers (11 [11%]). Physicians, psychologists, and other deliverers were involved to a lesser extent. 26 (26%) of these 101 evaluations explicitly stated that training was required for delivering the intervention.

#### Setting and mode of delivery

In terms of implementation settings, data reveal that the most promising evaluations were done in health-care facilities (27 [27%] of 101), followed by community centres (19 [19%]). Additionally, evaluations were conducted in participants’ homes (17 [17%] of 101), with a smaller proportion conducted online or remotely, such as evaluations through telephone calls (15 [15%] of 101). 55 (56%) of 101 evaluations were conducted in community settings and 46 (46%) in institutional settings. 67 (66%) of 101 evaluations were performed face to face, and 26 (26%) evaluations included components in a digital format, such as training modules or Adult Protective Services via teleconferencing. 36 (36%) of 101 evaluations were designed for group settings, and 21 (21%) were tailored for individual delivery; the remaining evaluations or descriptions (45 [45%]) used other implementation modes (eg, state-level legislation or self-guided books).

The duration of these evaluations ranged from less than 6 months to 30 months. Most of these evaluations (29 [29%] of 101) took between 0 and 6 months, followed by those that took between 13 and 24 months (ten [10%]). Seven (7%) of the 101 evaluations took between 7 and 12 months, and one evaluation took between 25 and 30 months. The remaining 54 (54%) intervention evaluations or descriptions did not specify the total duration of the intervention. The 11 evaluations that had a long total duration (ie, 13–30 months) were all conducted in HICs in Europe and North America, and nine (82%) of these 11 evaluations were conducted between 2004 and 2022.

#### Evaluation of outcomes

90 (89%) of the 101 intervention evaluations or descriptions provided comprehensive details on outcome evaluations.

Among these, 67 (74%) assessed one to three outcomes, focusing on reducing the occurrence of AOP, mitigating the associated risk factors, and enhancing efforts to detect and respond to abuse.

**Figure 3:**
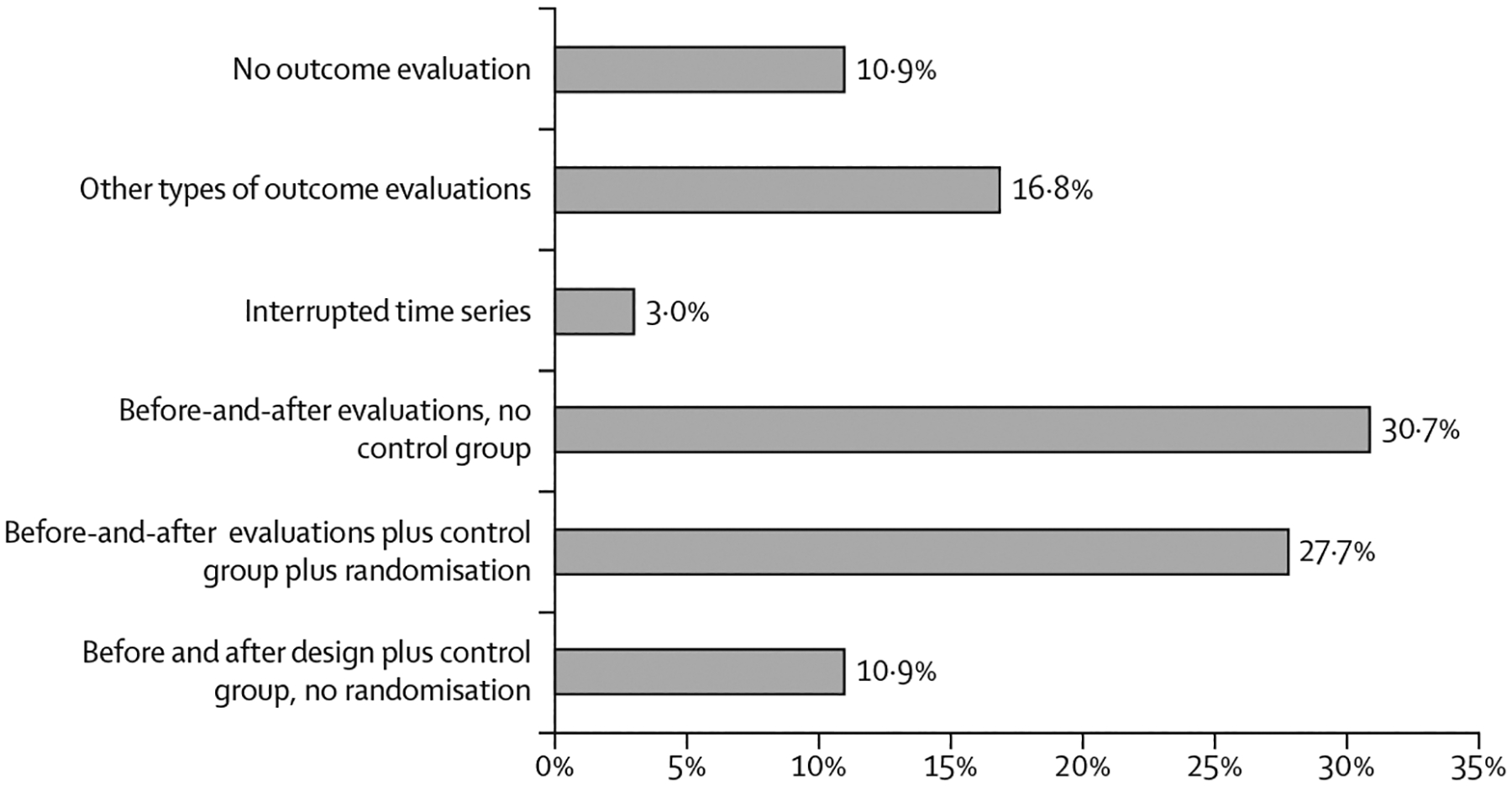
Study design used for outcome evaluation The results are given as a percentage of the 101 intervention evaluations or descriptions identified in this Review.

For evaluations using a causal inference research design (figure 3), the predominant design included baseline and follow-up evaluations without a control group (31 [31%] of 101), closely followed by randomised controlled trials (RCTs; 28 [28%]). Smaller proportions included before-and-after evaluations with a control group but without randomisation (11 [11%]) and interrupted time series (three [3%]). The remaining interventions adopted other types of outcome evaluations (17 [17%]), such as retrospective analysis or propensity score matching.

Considering that RCTs have the greatest methodological rigour, evaluations that used an RCT design (n=28) were compared with those that used other study designs (n=73). No substantial differences were observed across various parameters, such as study publication year, types of abuse targeted, actors involved, setting, and mode of delivery. However, when comparing the location in which evaluations were conducted, a substantial proportion of RCTs were conducted in the region of the Americas (11 [39%] of 28; all of these studies were conducted in the USA), with a similar proportion in the Eastern Mediterranean region (nine [32%] of 28), primarily in Iran. In contrast, most evaluations with other study designs were conducted in the region of the Americas (50 [69%] of 73), with a substantial concentration in the USA (42 [58%] of 73).

11 (11%) of the 101 intervention evaluations or descriptions had no outcome evaluation. However, these interventions were based on a plausible logic model, programme theory, theory of change, or some quantitative or qualitative empirical exploration of the approach, such as a qualitative case study.

## Discussion

As part of the initial phase of WHO’s AOP-IA project^8^ aimed to address the scarcity of evidence-based approaches to prevent and respond to AOP, a database was established to catalogue promising interventions. The database identified 89 unique interventions through a systematic four-step search strategy. The studies describing the interventions were published between 1993 and 2023, with nearly a third (n=35) categorised as original interventions. The interventions mainly targeted physical, psychological, and financial abuse and neglect. Most interventions were conducted in the region of the Americas, especially the USA, and focused primarily on victims or potential victims, particularly women. The interventions were predominantly administered by social workers and nurses across various settings such as health-care facilities, community centres, and homes. Face-to-face delivery was most common, and the effect of the intervention on outcomes was typically assessed through before-and-after designs, often incorporating control groups or randomisation.

Since 2010, a noticeable surge has been seen in publications on promising interventions, signalling increased interest in addressing AOP. This trend aligns with the global ageing population; projections indicate that by 2030, one in six people will be aged 60 years and older.^16^

Findings revealed a predominant focus of the interventions on immediate response rather than preventive measures, as secondary prevention outweighed primary prevention. Most of the interventions targeted physical, psychological, and financial abuse and neglect in similar proportions, and a smaller proportion addressed sexual abuse. The database developed from this study highlights only a small proportion of interventions that targeted systemic or organisational abuse, much of which probably occurs in residential care facilities.^17^ On the one hand, this observation is consistent with the small proportion of older people living in institutions worldwide (≤5% in HICs^18^). On the other, the small proportion of interventions that address systemic abuse could be a cause of concern given that 64·2% of professional caregivers in institutional settings have admitted to perpetrating AOP in the past year.^17^

Another aspect of concern is the observed geographical bias, with most interventions originating from HICs. Only 13 of the 101 intervention evaluations or descriptions originated in lower-middle-income countries and none in low-income countries, raising concerns about global inclusivity. To add to this concern, projections indicate that by 2050, 80% of older people will be living in low-income and middle-income countries (LMICs).^16^ The distribution of intervention evaluations or descriptions across country income levels is similar to that found in relation to interventions in health research and has been termed the 10/90 gap, as only 10% of research funding focuses on health problems that affect the poorest 90% of the global population.^19^ For instance, only 10% of outcome evaluations of interventions for interpersonal and self-directed violence or for child and adolescent mental health disorders were conducted in LMICs.^20,21^ Additionally, there were few interventions targeting AOP done in Latin America (only two in Cuba) and a notable absence of promising interventions from the African region. This observation could be attributed to either the search strategy that might not have captured interventions implemented in these regions or to the eligibility criteria that excluded them. Since no interventions were identified in the African region, a research team in Burkina Faso collaborating with this project conducted an additional review of promising interventions using an adapted search strategy, including search terms in French and modified eligibility criteria. This search revealed a tertiary community-based intervention aimed at supporting victims of witchcraft accusations in Burkina Faso. However, as this intervention was identified through a separate project conducted after the current one, the corresponding data have not been included in this analysis.

In terms of the delivery of interventions, more than half of the intervention evaluations or descriptions did not provide information on whether training was required for administration. Further, over half of the evaluations or descriptions were not based on a logic model, programme theory, or theory of change. Previous research has suggested that this issue could hinder the development of these interventions.^22^ However, the majority of the evaluations or descriptions of interventions (90 of 101 [89%]) had undergone some type of quantitative evaluation of effectiveness. Although over two-thirds of the evaluations did not include a control group with randomisation, thus raising concerns about the robustness of causal inferences and generalisability of findings, 28 interventions were evaluated using an RCT. Most evaluations (21 of 28 [75%]) that used an RCT design were conducted between 2013 and 2022.

The findings of this study suggest that the conclusion that no interventions addressing AOP have been effective, drawn in previous systematic reviews on the topic,^1–7^ might not be entirely accurate. Instead, several of the RCTs included in this Review are of high quality and have shown medium to large effects.^23–25^ Many factors could have contributed to the differing conclusions between the current study and previous systematic reviews. For instance, the narrow scope of inclusion years or the omission of the past few years in the previous systematic reviews, during which period the number of RCTs surged in the literature,^3,4,7^ might partly account for these differences.

This Review has some notable strengths. First, the Review is based on extensive and systematic searches across multiple sources, with no language or publication year restrictions, including an international call for expert suggestions on interventions. Second, the interventions were identified after a rigorous screening and coding process. Finally, by providing detailed and standardised information on 89 promising interventions reported in 101 evaluations or descriptions of interventions designed to prevent and respond to AOP, the database developed in this study serves as a rich repository of evidence-based interventions that could inform and advance future research and practice in the field.

However, several limitations of this study should also be acknowledged. First, since the aim of the Review was to identify promising interventions, we excluded evaluations with null or negative findings in all the outcomes examined, although we maintained a record of these evaluations. Second, we did not conduct a meta-analysis. The goal of this study was to establish an intervention accelerator to advance the development of effective interventions for preventing AOP across HICs and LMICs. Given this aim and the constraints with respect to available resources and time, we chose to focus on identifying and providing an overview of these promising interventions. Third, although efforts were made to include interventions from different contexts and our selection criteria did not have any language restrictions, our search strategy, which was formulated in English, might have identified fewer interventions from specific regions of the world.

In conclusion, this Review highlights the need for high-quality evaluations of promising interventions to address AOP in low-income countries. In addition, existing systematic reviews need to be updated to include and synthesise the findings from all 28 RCTs with positive findings included in the database developed in this study.

## Supplementary Material

Campo-Tena et al Appendix
