# Supplementary material for: Intervention accelerator to prevent and respond to abuse of older people: insights from key promising interventions
Source: Lancet Healthy Longev. Author manuscript; Available in PMC 2024 Dec 30. (PMC11682911; doi:10.1016/j.lanhl.2024.100647)

Table of Contents

Appendix 1. Criteria for Screening of Promising Interventions 2

Appendix 2. Coding Sheet for Data Extraction for Promising Interventions from Mega-Map and from Updated Searches for Primary Studies 9

Appendix 3. List of Publications and Interventions Included. 16

Appendix 4. Publication Year of the Promising Interventions (101 intervention evaluations or descriptions).. 27

Appendix 5. Number of Promising Interventions to Address Abuse of Older People Per Country (n = 101). 28

Appendix 1. Criteria for Screening of Promising Interventions

	Inclusion	Exclusion	Definitions/explanation
Type of study	• Primary study consisting of an outcome. evaluation of an intervention to prevent or respond to abuse of older people.	• Reviews of multiple studies of any sort (e.g., systematic review, narrative review, meta-analysis, etc.) & protocols.	
Beneficiaries of Intervention	• Victims of abuse of older people (i.e., people 60 years and over).<br><br>• If the population of intervention beneficiaries includes victims of abuse of older people (60+) or of intimate partner violence (who are 60+), but also includes other age groups, the relevant findings must be available in a disaggregated form for people 60+ (i.e., findings for people 60+ must be presented separately).<br><br>• If the age cut-off is less than 60 (e.g., 55) but 50 or more and the mean age of the sample is over 65, then the study should be included, even if findings are not disaggregated for those 60+.	• Any population group that does not meet the inclusion criteria, i.e.,<br><br>o Population includes people under the age of 60 and findings for those 60+ are not disaggregated; AND<br><br>o If age cut-off <60 but 50 or more, mean age is 65 or less.	Abuse of older people:<br><br>• Definition of abuse of older people/elder abuse: a single or repeated act or lack of appropriate action, occurring within any relationship **where there is an expectation of trust** which causes harm or distress to an older person (i.e., 60+). The main forms of elder abuse generally recognized, and which can occur in the community and institutional settings are physical, psychological, financial/material, sexual abuse and neglect, systemic/organizational abuse, and poly-victimization.<br><br>• This definition of abuse of older people includes intimate partner violence perpetrated against a person 60 years or older.
Intervention Participants	• Populations which the interventions to prevent or respond to the **abuse of older people** are aimed at including:<br><br>o Perpetrators of abuse of older people (of any age, e.g., spouse/intimate partner, family, neighbours, non-professional and professional caregivers).<br><br>o Victims of abuse of older people (i.e., people 60 years and over).	• Any population group perpetrating violence, crime or abuse against a person 60+ where there is no relationships in which there is an expectation of trust. This means, for instance, that interventions aiming to reduce older people's victimization in street crime perpetrated by strangers, etc. are excluded.	Intervention participants:<br><br>• Individuals or groups who are directly involved in the intervention program or study. These participants are selected based on specific criteria relevant to the intervention's goals and objectives. The intervention participants can vary depending on the nature of the intervention and its target population. In the context of interventions to prevent or respond to the abuse of older people, the intervention participants can encompass a broad range of individuals, including perpetrators, older people, formal and informal caregivers, the

	Inclusion	Exclusion	Definitions/explanation
	o “Concerned others”, i.e., individuals who are involved in the situation but have not perpetrated the abuse, such as friends, neighbours, and relatives of the victim of abuse of older people (of any age).<br><br>o Non-professional caregivers at risk of perpetrating abuse of older people (of any age, e.g., spouses/intimate partners, family, friends, neighbours, volunteers, etc.).<br><br>o Professional caregivers at risk of perpetrating abuse of older people (of any age, e.g., staff in long-term care institutions, agency caregivers, nurses, social workers, physicians, administrators or managers for organisational/institutional abuse, etc.).<br><br>o Non-caregivers at risk of perpetrating abuse of older people (of any age, e.g., spouse/intimate partner, family, friends, acquaintances, neighbours, etc.).<br><br>o General population (of any age, e.g., awareness campaigns, community level interventions, system level interventions such as laws and policies).<br><br>o Institutions themselves (rules and regulations governing care home, long term care facilities, hospitals, banks, etc.).		general population, and the institutions themselves (see inclusion criteria in this row).

	Inclusion	Exclusion	Definitions/explanation
Type of abuse	• Physical.<br>• Psychological (verbal/emotional), including accusations of witchcraft.<br>• Intimate partner violence, including psychological and physical abuse.<br>• Sexual (by any perpetrator).<br>• Financial, including scams and fraud.<br>• Neglect (including abandonment).<br>• Systemic/organizational/or institutional abuse.<br>• Resident-to-resident abuse.	• Self-neglect.<br>• Ageism (e.g., stereotypes, prejudice, and discrimination based on age).<br>• The use of restraints (physical & chemical) and seclusion.	• If an intervention aims both to prevent or respond to abuse of older people and other forms of violence (e.g., intimate partner violence in younger age groups) it will be included, provided findings are disaggregated for those 60+ (when relevant).<br>• We will consider that financial/material abuse includes scams and frauds in as much as the older person has an expectation of trust – even if misplaced – in relation to the person the scammer or fraudster is purporting to be.
Intervention	• **All interventions** (i.e., primary, secondary, or tertiary prevention; or universal, selective/ed, and indicated) aiming to prevent or respond to **abuse of older people.**	Interventions that exclusively aim to address one or a combination of the following:<br>• Validation studies of tools to screen/identify/detect abuse of older people.<br>• Ageism (e.g., stereotypes, prejudice, and discrimination based on age).	Intervention and intervention types<br>• The definition of i**ntervention** we will use will follow that of a "health intervention" which refers to an act performed for, with or on behalf of a person or a population whose purpose is to assess, improve, maintain, promote or modify health, functioning or health conditions. Types of interventions vary widely and range from narrowly focused interventions such as one-to-one psychological treatments to much broader interventions such as public health laws and policies.<br>• **Primary, secondary, and tertiary prevention** interventions will be included:<br>• Primary prevention – approaches that aim to prevent abuse before it occurs.<br>• Secondary prevention – approaches that focus on the more immediate responses to abuse, such as pre-hospital care, emergency services or treatment for sexually transmitted diseases following a rape.

	Inclusion	Exclusion	Definitions/explanation
			• Tertiary prevention – approaches that focus on long-term care in the wake of abuse, such as rehabilitation of perpetrators and attempts to lessen trauma or reduce the long-term harms and disability associated with abuse in victims.<br>• **Universal, selective, and indicated interventions** will be included:<br>• Universal interventions – approaches aimed at groups or the general population without regard to individual risk; examples include media awareness campaigns delivered to the whole population.<br>• Selected interventions – approaches aimed at those considered at heightened risk for abuse (having one or more risk factors for abuse); an example of such an intervention is training for staff in long-term care institutions about abuse of older people.<br>• Indicated interventions – approaches aimed at those who have already demonstrated abusive behavior, such as treatment for perpetrators of abuse, to prevent recurrence; or those who have been victims of abuse to mitigate impact of abuse.
Setting	• All settings, including:<br>o Community.<br>o Institutions (e.g., long-term care institutions, hospitals, prisons or justice, etc.).<br>o Any others.		
Evaluation	• Intervention has been evaluated using a quantitative research design for causal inference and has been found to have a statistically significant positive impact on at least one outcome of interest (see outcomes	• Intervention has been evaluated using any kind of quantitative research design for causal inference and has been found to have no statistically significant effect or a	• Quantitative research designs for causal inference include, for instance, one group before-and-after evaluations (i.e., with no control group), pre-test/post-test with control group (whether or not randomized), interrupted time series, regression discontinuity designs, etc.

	Inclusion	Exclusion	Definitions/explanation
	below), even if findings for other outcomes are null or negative.<br><br>● Also include if quantitative research design for causal inference is part of a mixed design.<br><br>OR<br><br>● Intervention has not been evaluated but is based on a plausible logic model, programme theory, theory of change which is described in detail (not just referenced or alluded to) and some quantitative or qualitative empirical exploration of approach has been carried out (such as a pilot test or a process or formative evaluation).<br><br>OR<br><br>● Intervention has been evaluated in a previous study and found to have a statistically significant positive impact on at least one outcome of interest (see outcomes below), even if findings for other outcomes are null or negative. In this case, please take note of previous study so we can include.	negative effect on any of the outcomes (see outcomes of interest below).<br><br>● Intervention has been "evaluated" using a qualitative research design or based on expert opinion or satisfaction of intervention participants <u>and</u> is not based on a based on a plausible logic model, programme theory, or theory of change which is described in some detail.<br><br>● However, evaluation of intervention based solely on expert opinion, whether or not it is based on a plausible logic model, programme theory, or theory of change will be excluded.	
Outcomes of evaluation	● Reductions in the occurrence or the severity of abuse of older people.<br><br>● Reduction in risk factors associated with abuse of older people (unless actual abuse is measured and findings are either null or statistically significantly negative).	● If measure of satisfaction of participants in interventions is the only outcome measured.<br><br>● Opinion of experts on purported effectiveness of intervention.	● See types of abuse listed above.

	Inclusion	Exclusion	Definitions/explanation
	• Increase in protective factors associated with abuse of older people.<br>• Increased efforts to identify/detect, report and respond to abuse of older people.<br>• Increased awareness and knowledge of abuse of older people.<br>• Increased support for people who are the target of abuse of older people and for "concerned others" or perpetrators.<br>• Reduction in psychological distress (depression, anxiety, PTSD, or other mental health measures) associated with abuse of older people.<br>• Financial recovery, security, protection (especially for financial abuse).		
Time period for development/ implementation /evaluation of intervention	• Any<br>• The study/description of intervention can have been published at any time and the interventions can have been implemented at any time. No time restrictions apply.	• None	
Geographical area, region, country income level in which intervention implemented.	• Any.<br>• Countries from all regions and income levels will be included.	• None	

	Inclusion	Exclusion	Definitions/explanation
Language of report/paper	• Any. The study/description of intervention can be in any language. No language restrictions will be applied.	• None	
Additional information<br><br>If there is not enough information in the report or paper to apply one or more criteria, then please indicate this in the appropriate column in the spreadsheet.			

Appendix 2. Coding Sheet for Data Extraction for Promising Interventions from Mega-Map and from Updated Searches for Primary Studies

#	Item	Description	Comment
1.	Brief name	Provide the name or a phrase that describes the intervention.	
2.	Source of intervention	▪ Mega-map (i.e., primary study on intervention included in mega-map);<br>▪ International call;<br>▪ Updated searches for systematic review of interventions to prevent and respond to abuse of older people.	This item is for internal use only
3.	Key people involved	▪ For each key person:<br>o Specify role of key person, as much as possible;<br>o Provide:<br>▪ First and family name;<br>▪ Contact details (if available):<br>● Name of institution;<br>o Don't know or not specified.<br>● Address;<br>o Don't know or not specified.<br>● E-mail.<br>o Don't know or not specified.	Key people include, e.g., developer of original intervention, evaluator of intervention, adaptor of existing intervention, etc.;
4.	Adaptation of previous intervention	Was this an adaptation or modification of a previous intervention?<br>- Yes.<br>- No, explicitly stated that this is the original intervention.<br>- Don't know or not specified.<br><br>If yes, which intervention (please specify name or phrase that describes intervention and provide references, if available).	
5.	Aim of intervention	Describe the stated aim of the intervention.	
6.	Type of abuse	Select type(s) of abuse of older people the intervention aims to address:<br>● Any<br>● Physical;<br>● Psychological (verbal/emotional), including accusations of witchcraft;<br>● Sexual;	

#	Item	Description	Comment
		● Financial, including scams and fraud;<br>● Neglect, including abandonment;<br>● Systemic/organizational/or institutional abuse;<br>▪ Poly-victimization (i.e., more than one of the types above);<br>▪ Intimate partner violence (if victim 60+);<br>▪ Resident-to-resident (e.g., in long-term care institutions);<br>▪ Other, please specify	If “poly-victimization” is selected, also specify the individual types of abuse this covers by checking individual types list (if this information is available).
7.	Who is the intervention being delivered to?	Which population is this intervention being delivered to:<br>▪ Perpetrators of abuse of older people (of any age)<br>o If possible, specify type of perpetrator (e.g., spouse/intimate partner, family, neighbours, non-profession and professional caregivers);<br>▪ Potential victims of abuse of older people (i.e., people 60 years and over);<br>▪ “Concerned others” (of any age)<br>o If possible, specify type of “concerned other” (e.g., individuals who are involved in the situation but have not perpetrated the abuse, such as friends, neighbours, and relatives of the victim of abuse of older people);<br>▪ Non-professional caregivers at risk of perpetrating abuse of older people (of any age)<br>o If possible, specify type of non-professional caregivers (e.g., spouse/intimate partner, family, friend, neighbour, volunteer, etc.);<br>▪ Professional caregivers at risk of perpetrating abuse of older people (of any age)<br>o If possible, specify type of professional caregiver (i.e., their profession/occupation, e.g., staff in long-term care institutions, agency caregivers, nurses, social workers, physicians, etc.);<br>▪ Non-caregivers at risk of perpetrating abuse of older people (of any age)<br>o If possible, specify type of non-caregiver (e.g., spouse/intimate partner, family, friends, acquaintances, neighbours, etc.) (of any age);<br>▪ General population<br>o If possible, specify sub-set of general population (of any age) (e.g., for awareness campaigns, community level intervention, system level interventions, such as a laws and policies);	

#	Item	Description	Comment
		▪ Institutions themselves (rules and regulations governing care home, long term care facilities, hospitals, banks, etc.)<br>o Specify type of institution;<br>▪ Other, please specify.	
8.	Other characteristics of population intervention is being delivered to, if available	▪ Gender (proportion in sample);<br>▪ Age (range, mean, etc.).<br>▪ Education level (None at all; Incomplete primary education; Primary School (5 or 6 years total); Secondary school (8-10 years total); Senior Secondary/High school (11-12 years total); Vocational/Technical School (10 - 12 years total); University Undergraduate Degree; University Master’s degree; University Doctoral Degree; Other. Please specify: ____<br>▪ Any information on socioeconomic status. Please specify:	
9.	Age of beneficiary population (i.e., older people 60+ at risk of or experiencing abuse)	▪ Please provide the following data, if available:<br>o Age cut-off;<br>o Mean age;<br>o Age range.	
10.	Who is this intervention being delivered by?	Which professional group is delivering this intervention?<br>▪ Psychologists;<br>▪ Social workers;<br>▪ Nurses;<br>▪ Physicians;<br>▪ Other healthcare workers, please specify;<br>▪ Educators;<br>▪ Peers;<br>▪ Volunteers;<br>▪ Other, please specify;<br>▪ Not applicable because it is a systems level intervention (e.g., campaign, policy, law, etc.).<br>▪ Don’t know or not specified.	
11.	Setting	Which kind of setting is the intervention being delivered in?<br>▪ Healthcare facility;<br>▪ Educational institution;<br>▪ Community center;<br>▪ Home-based/home-visiting;<br>▪ Home-based/home-visiting;<br>▪ Online/remotely;	

#	Item	Description	Comment
		▪ Other, please specify;<br>▪ Don't know or not specified.<br><br>Describe any relevant features of the setting, including infrastructure required.	
12.	Training	Is any training required to deliver this intervention?<br>▪ Yes<br>▪ No<br>▪ Not specified or don't know<br><br>If yes, what kind and how much (hours, days, weeks, etc.)?	
13.	Theory	a) Is the intervention explicitly based on some kind of theory (programme theory, logic model, theory of change, etc.)<br>☐ Yes<br>☐ No<br>b) If yes, provide name or a brief phrase that describes theory;<br>c) If yes, how much detail is provided about this theory:<br>▪ Only referred to briefly;<br>▪ Explained in some detail (i.e., ~one paragraph)<br>▪ Explained in great detail (i.e., more than one paragraph and/or diagram);<br>d) If yes, cut and paste whatever information is available on this theory	
14.	Materials	Describe briefly any physical or informational materials used in the intervention, including those provided to participants or used in intervention delivery or in training of intervention providers.<br>Provide any information on where the materials can be accessed (e.g., online appendix, URL).<br>▪ Don't know or not specified.	
15.	Procedures	Describe briefly each of the procedures, activities, and/or processes used in the intervention, including any enabling, empowerment, or support activities.	
16.	Mode of delivery	What is the mode of delivery of the intervention? (you can select more than one)	

#	Item	Description	Comment
		▪ Face-to-face, or<br>▪ Digital;<br><br>▪ Group, or<br>▪ Individual;<br><br>▪ Other (e.g., public awareness campaign aimed at community/larger population), please specify:<br>▪ Don't know or not specified.	
17.	Location - country	Country/ies intervention has been delivered in:<br>● Don't know or not specified.	If more than one, list them all
	Location - city-sub-national entity	For each country, please specify city-ies or province-s/state-s/district-s (i.e., sub-national entity) intervention has been delivered in:<br>● Don't know or not specified.	
	Location – urban/rural	● Urban<br>● Rural<br>● Don't know or not specified	
18.	When and how	- When was the intervention delivered (month/year)<br>- Don't know or not specified.<br>- Number of sessions<br>- Don't know or not specified.<br>- Schedule (e.g., daily, weekly, 3x week, monthly, etc.);<br>- Don't know or not specified.<br>Duration of each session (minutes);<br>- Don't know or not specified.<br>- Other, please specify	

#	Item	Description	Comment
19.	Process evaluation	Did they assess how well the intervention was implemented (i.e., intervention adherence or fidelity)?<br>▪ Yes<br>▪ No<br>If yes, describe how and by whom.<br>And if any strategies were used to maintain or improve fidelity, describe them.	
20.	Outcome evaluation	Did they evaluate if the intervention had the desired outcome(s)?<br>▪ Yes<br>▪ No<br>● Don’t know or not specified.<br><br>What kind of research design for causal inference was used for this outcome evaluation<br>▪ Before-and-after with no control group;<br>▪ Before-and-after with control group, but without randomization (including with efforts to make experimental and control groups more similar, such as matched design, propensity score matching);<br>▪ Before-and-after with control group, with randomization (i.e., RCT, including cluster randomized studies);<br>▪ Interrupted time series;<br>▪ Other research design for causal inference, please specify:<br><br>What were the outcomes measured that are relevant to abuse of older people?<br><br>What were the main findings for each of the main outcomes relevant to the abuse of older people that were measured?<br>▪ Positive (i.e., statistically significant effect in desired direction);<br>▪ Negative (i.e., statistically significant effect in direction opposite to that of desired direction);<br>▪ Null (no statistically significant effect).<br>▪ Don’t know or not specified.<br><br>If no outcome evaluation using a research design for causal inference was carried out, were one of the following types of studies carried out on the intervention?<br>▪ A qualitative study	If more than one evaluation has been conducted, please list all outcome evaluations with references if available.

#	Item	Description	Comment
		▪ Study based on expert opinion<br>▪ Participant satisfaction survey<br>▪ One or more case studies<br>▪ Other type of study, please specify.<br>▪ Don't know or not specified.	
21	Any other relevant information (e.g., feasibility study, pilot evaluation, etc.).		

Appendix 3. List of Publications and Interventions Included

Number of interventions evaluated/described meeting inclusion criteria (N=101)	Publications (N=99)	Interventions (N=89)
1	Alon, S., & Berg-Warman, A. (2014). Treatment and prevention of elder abuse and neglect: Where knowledge and practice meet—A model for intervention to prevent and treat elder abuse in Israel. *Journal of elder abuse & neglect*, *26*(2), 150-171.	Model for Intervention to Preventand Treat Elder Abuse in Israel
1	Alon, S., Lang, B., & Band-Winterstein, T. (2022). Do training programs promote identification and treatment in cases of elder abuse in Long Term Care (LTC) facilities? *Educational Gerontology, 48*(8), 355-367.	Specialized Unit for the Prevention and Treatment of Elder Abuse (SUPTEA)
1	Anetzberger, G. J., Dyer, C. B., Barth, J., Portal, B., Hyman, D. J., Pavlik, V. N., . . . Gleason, M. S. (1999). A case series of abused or neglected elders treated by an interdisciplinary geriatric team. *Journal of elder abuse & neglect, 10*(3-4), 131-139.	Interdisciplinary Geriatric Assessment and Intervention Teams (IGAIT)
1	Anetzberger, G. J., Palmisano, B. R., Sanders, M., Bass, D., Dayton, C., Eckert, S., & Schimer, M. R. (2000). A model intervention for elder abuse and dementia. *The Gerontologist, 40*(4), 492-497.	A Model Intervention for Elder Abuse and Dementia
1	Bartels, S. J., Miles, K. M., Van Citters, A. D., Forester, B. P., Cohen, M. J., & Xie, H. (2005). Improving mental health assessment and service planning practices for older adults: a controlled comparison study. *Mental Health Services Research, 7*, 213-223.	Practice change intervention/ quality improvement toolkit/ decision-support process
1	Beaulieu, M., Côté, M., & Diaz, L. (2017). Police and partners: New ways of working together in Montréal. *The Journal of Adult Protection, 19*(6), 406-417.	Integrated Police Response for Abused Seniors (IPRAS)
1	Brownell, P., & Heiser, D. (2013). Psycho-educational support groups for older women victims of family mistreatment: A pilot study. In *Elder Abuse and Mistreatment* (pp. 145-160): Routledge.	Psycho-educational support group
1	Brownell, P., & Wolden, A. (2003). Elder abuse intervention strategies: Social service or criminal justice? *Journal of Gerontological Social Work, 40*(1-2), 83-100.	Comparison between the social service intervention and criminal justice intervention
1	Brymer, C., Cormack, C., & Spezowka, K.-A. (1998). Improving the care of the elderly in a rural county through education. *Gerontology & Geriatrics Education, 19*(1), 55-64.	Modified Geriatric Educational Needs Assessment (GENA)

Number of interventions evaluated/described meeting inclusion criteria (N=101)	Publications (N=99)	Interventions (N=89)
1	Buckwalter, K. C., Campbell, J., Gerdner, L. A., & Garand, L. (1996). Elder mistreatment among rural family caregivers of persons with Alzheimer's disease and related disorders. *Journal of Family Nursing, 2*(3), 249-265.	A multisite training project
1	Burnes, D., MacNeil, A., Connolly, M.-T., Salvo, E., Kimball, P. F., Rogers, G., & Lewis, S. (2022). A qualitative evaluation of the “RISE” elder abuse intervention from the perspective of adult protective services caseworkers: Addressing a service system gap. *Journal of elder abuse & neglect, 34*(5), 329-348.	Relational, Individual, Social, and Environmental levels of ecological influence (RISE)
1	Burnett, J., Dyer, C. B., Clark, L. E., & Halphen, J. M. (2019). A statewide elder mistreatment virtual assessment program: Preliminary data. *Journal of the American Geriatrics Society, 67*(1), 151-155.	TEAM-FACN (Texas Elder Abuse and Mistreatment Institute-Forensic Assessment Center Network)
1	Butler, L., & Manthorpe, J. (2016). Putting people at the centre: facilitating Making Safeguarding Personal approaches in the context of the Care Act 2014. *The Journal of Adult Protection, 18*(4), 204-213.	Making Safeguarding Personal (MSP)
1	Cooper, A., Lawson, J., Lewis, S., & Williams, C. (2015). Making safeguarding personal: learning and messages from the 2013/14 programme. *The Journal of Adult Protection, 17*(3), 153-165.	Making Safeguarding Personal (MSP)
1		
1	Carey, C., Hodges, J., & Webb, J. K. (2018). Changes in state legislation and the impacts on elder financial fraud and exploitation. *Journal of elder abuse & neglect, 30*(4), 309-319.	State legislation on elder financial fraud and exploitation (EFFE)
1	Cripps, D. (2001). Rights focused advocacy and elder abuse. *Australasian Journal on Ageing, 20*(1), 17-22.	Rights Focused Advocacy Model
1	Davis, R. C., Medina, J., & Avitabile, N. (2001). Reducing repeat incidents of elder abuse: Results of a randomized experiment: Final report. *New York, NY: US Department of Justice*.	Public education intervention & Follow up home visits
1	DePrince, A. P., Hasche, L. K., Olomi, J. M., Wright, N. M., & Labus, J. (2019). A randomized-control trial testing the impact of a multidisciplinary team response to older adult maltreatment. *Journal of elder abuse & neglect, 31*(4-5), 307-324.	Multidisciplinary Team

Number of interventions evaluated/described meeting inclusion criteria (N=101)	Publications (N=99)	Interventions (N=89)
1	Despaigne Vinent, M., Jiménez Betancourt, E., & Martínez Despaigne, B. (2011). Intervención educativa sobre violencia a integrantes de la Universidad del Adulto Mayor" 24 de Febrero". *Medisan, 15*(1), 105-111.	Educational intervention on violence for members of the "24 de Febrero" Older Adult University
1	Désy, P. M., & Prohaska, T. R. (2008). The geriatric emergency nursing education (GENE) course: an evaluation. *Journal of Emergency Nursing, 34*(5), 396-402.	Geriatric Emergency Nursing Education (GENE)
1	Dianati, M., Azizi-Fini, I., Oghalaee, Z., Gilasi, H., & Savari, F. (2019). The impacts of nursing staff education on perceived abuse among hospitalized elderly people: a field trial. *Nursing and Midwifery Studies, 8*(3), 149-154.	Reducing Perceived Abuse in Hospital
1	Dkhar, E., Balu, V., & Kamei, S. (2022). Effectiveness of educational programme on knowledge regarding abuse and age-related changes among elderly population. *J Clin Diagn Res, 16*, LC13-18.	Educational programme on knowledge regarding abuse and age-related changes among the elderly.\
1	Drossel, C., Fisher, J. E., & Mercer, V. (2011). A DBT skills training group for family caregivers of persons with dementia. *Behavior therapy, 42*(1), 109-119.	Dialectical Behavior Therapy (DBT)
1	Du Mont, J., Kosa, D., Yang, R., Solomon, S., & Macdonald, S. (2017). Determining the effectiveness of an Elder Abuse Nurse Examiner Curriculum: A pilot study. *Nurse Education Today, 55*, 71-76.	Elder Abuse Nurse Examiner Curriculum
1	Du Mont, J., Kosa, S. D., Kia, H., Spencer, C., Yaffe, M., & Macdonald, S. (2020). Development and evaluation of a social inclusion framework for a comprehensive hospital-based elder abuse intervention. *PLoS one, 15*(6), e0234195.	Social inclusion framework
1	Ejaz, F. K., Rose, M., & Anetzberger, G. (2017). Development and implementation of online training modules on abuse, neglect, and exploitation. *Journal of elder abuse & neglect, 29*(2-3), 73-101.	Online training modules on elder abuse, neglect, and exploitation
1	Elsherbiny, M. M. K., & Al Maamari, R. H. (2018). The effectiveness of logotherapy in mitigating the social isolation of neglected institutionalised older people. *British Journal of Social Work, 48*(4), 1090-1108.	Logotherapy
1	Elvegård, K.-I. L., Olsen, T., Tøssebro, J., & Paulsen, V. (2020). TryggEst: Bedre beskyttelse av overgrepsutsatte voksne: Sluttrapport: To års forsøk med TryggEst.	TryggEst
1	Ernst, J. S., & Smith, C. A. (2012). Assessment in Adult Protective Services: do multidisciplinary teams make a difference? *Journal of Gerontological Social Work, 55*(1), 21-38.	Multidisciplinary APS Teams

Number of interventions evaluated/described meeting inclusion criteria (N=101)	Publications (N=99)	Interventions (N=89)
1	Estebsari, F., Dastoorpoor, M., Mostafaei, D., Khanjani, N., Khalifehkandi, Z. R., Foroushani, A. R., . . . Taghdisi, M. H. (2018). Design and implementation of an empowerment model to prevent elder abuse: a randomized controlled trial. *Clinical interventions in aging*, 669-679.	Empowerment Education
1	Evanina, K. (2014). *The effect of education on elder abuse.* Duquesne University,	Competence with Compassion: A Universal Core Curriculum
1	Ezalina, E., Machmud, R., Effendi, N., & Maputra, Y. (2019). Effectiveness of the elderly caring model as an intervention to prevent the neglect of the elderly in the family. *Open access Macedonian journal of medical sciences, 7*(14), 2365.	Elderly Caring Model Preventing Neglect
1	Fernández, S. D., Pérez, Y. M. C., & Fernández, B. C. D. (2012). Programa de intervención educativa para promover conductas asertivas en el adulto mayor. *Mediciego, 18*(2).	Programa de Intervención Educativa
1	Filinson, R. (1993). An evaluation of a program of volunteer advocates for elder abuse victims. *Journal of elder abuse & neglect*, *5*(1), 77-94.	Elder Abuse Support Project
1	Fisher, J. M., & Walker, R. W. (2014). A new age approach to an age old problem: using simulation to teach geriatric medicine to medical students. *Age and ageing, 43*(3), 424-428.	Simulation-based geriatric medical training
1	Gassoumis, Z. D., Navarro, A. E., & Wilber, K. H. (2015). Protecting victims of elder financial exploitation: The role of an elder abuse forensic center in referring victims for conservatorship. *Aging & Mental Health*, *19*(9), 790-798.	Los Angeles County Elder Abuse Forensic Center
1	Navarro, A. E., Gassoumis, Z. D., & Wilber, K. H. (2013). Holding abusers accountable: An elder abuse forensic center increases criminal prosecution of financial exploitation. *The Gerontologist*, *53*(2), 303-312.	Los Angeles County Elder Abuse Forensic Center
1	Navarro, A. E., Wilber, K. H., Yonashiro, J., & Homeier, D. C. (2010). Do we really need another meeting? Lessons from the Los Angeles county elder abuse forensic center. *The Gerontologist, 50*(5), 702-711.	Los Angeles County Elder Abuse Forensic Center
1	Ghaffari, F., Alipour, A., & Fotokian, Z. (2020). The effects of education on nurses' ability to recognize elder abuse induced by family members. *Nursing and Midwifery Studies, 9*(1), 1-8.	Educational Program on Nurses' Ability to Recognize EA

**Number of interventions evaluated/described meeting inclusion criteria (N=101)**	**Publications (N=99)**	**Interventions (N=89)**
3	Hafford, C., Nguyen, K., & Henning, S. (2016). An evaluation of AoA's program to prevent elder abuse. *Final report. Prepared for Office of Disability, Aging and Long-Term Care Policy Office of the Assistant Secretary for Planning and Evaluation, US Department of Health and Human Service. NORC at the University of Chicago*.	Take AIM; Critical Time Intervention (CTI); Enhanced Multi-Disciplinary Team (E-MDT)
1	Halarewicz, A., Gelman, C., Ghesquiere, A., & Rogers, G. (2019). "Opening the door": developing and pilot testing an adult protective services worker engagement training in New York city. *Journal of elder abuse & neglect, 31*(3), 191-208.	Worker Engagement Training - Pilot in New York City
1	Hall, L., Campbell, R., Gross, E., & Lichtenberg, P. A. (2022). The impact of financial coaching on older adult victims of financial exploitation: A quasi-experimental research study. *Financial counseling and planning: the journal of the Association for Financial Counseling and Planning Education, 33*(1), 66.	The successful aging through financial empowerment (SAFE) program
1	Lichtenberg, P. A., Hall, L., Gross, E., & Campbell, R. (2019). Providing assistance for older adult financial exploitation victims: Implications for clinical gerontologists. *Clinical Gerontologist, 42*(4), 435-443.	Assistance on Financial Exploitation Victims
1	Harmer-Beem, M. (2005). The perceived likelihood of dental hygienists to report abuse before and after a training program. *American Dental Hygienists' Association, 79*(1), 7-7.	PANDA (Prevent Abuse and Neglect through Dental Awareness)
1	Harries, P., Davies, M., Gilhooly, K., Gilhooly, M., & Tomlinson, C. (2014). Educating novice practitioners to detect elder financial abuse: a randomised controlled trial. *BMC medical education, 14,* 1-9.	Decision-training on Financial Elder Abuse
1	Harries, P., Yang, H., Davies, M., Gilhooly, M., Gilhooly, K., & Thompson, C. (2014). Identifying and enhancing risk thresholds in the detection of elder financial abuse: a signal detection analysis of professionals' decision making. *BMC medical education, 14*, 1-12.	Signal Detection for Elder Financial Abuse
1	Hayslip Jr, B., Reinberg, J., & Williams, J. (2015). The impact of elder abuse education on young adults. *Journal of elder abuse & neglect, 27*(3), 233-253.	Elder Abuse Education For Young Adults
1	Hazrati, M., Hamid, T. A., Ibrahim, R., Hassan, S. A., Sharif, F., & Bagheri, Z. (2017). The dyadic effects of controlling emotional abuse on subjective emotional	Emotional Focused Psycho Educational Intervention (EFPEI)

Number of interventions evaluated/described meeting inclusion criteria (N=101)	Publications (N=99)	Interventions (N=89)
	experience in Iranian older married couples. *Australian and New Zealand Journal of Family Therapy, 38*(3), 514-528.	
1	Hazrati, M., Hamid, T. A., Ibrahim, R., Hassan, S. A., Sharif, F., & Bagheri, Z. (2017). The effect of emotional focused intervention on spousal emotional abuse and marital satisfaction among elderly married couples: A randomized controlled trial. *International Journal of Community Based Nursing and Midwifery, 5*(4), 329.	Emotional Focused Intervention
1	Hernandez-Tejada, M. A., Skojec, T., Frook, G., Steedley, M., & Davidson, T. M. (2021). Addressing the psychological impact of elder mistreatment: Community-based training partnerships and telehealth-delivered interventions. *Journal of elder abuse & neglect, 33*(1), 96-106.	Telehealth
1	Holkup, P. A., Salois, E. M., Tripp-Reimer, T., & Weinert, C. (2007). Drawing on wisdom from the past: an elder abuse intervention with tribal communities. *The Gerontologist, 47*(2), 248-254.	Family Care Conference
1	Hsieh, H.-F., Wang, J.-J., Yen, M., & Liu, T.-T. (2009). Educational support group in changing caregivers' psychological elder abuse behavior toward caring for institutionalized elders. *Advances in health sciences education, 14,* 377-386.	Educational Support Groups for Caregivers
1	Khanlary, Z., Maarefvand, M., Biglarian, A., & Heravi-Karimooi, M. (2016). The effect of a family-based intervention with a cognitive-behavioral approach on elder abuse. *Journal of elder abuse & neglect, 28*(2), 114-126.	Family-Based Cognitive-Behavioral Social Work (FBCBSW)
1	Kosa, S. D., Du Mont, J., & Macdonald, S. (2020). Development and evaluation of an elder abuse forensic nurse examiner e-learning curriculum. *Gerontology and geriatric medicine, 6*, 2333721420965819.	EA Nurse Examiner e-Learning Curriculum
1	Liu, P.-J., Hass, Z., Stratton, S. K., Conrad, K. M., & Conrad, K. J. (2022). Examining adult protective services outcomes: Services associated with the decrease of mistreatment differed by elder mistreatment type. *The Gerontologist, 62*(9), 1359-1368.	Adult protective services, services associated with mistreatment reduction
1	Ludvigsson, M., Motamedi, A., Westerlind, B., Swahnberg, K., & Simmons, J. (2022). Responding to Elder Abuse in GERiAtric care (REAGERA) educational intervention for healthcare providers: a non-randomised stepped wedge trial. *BMJ open, 12*(5), e060314.	Responding to Elder Abuse in GERiAtric care (REAGERA)

Number of interventions evaluated/described meeting inclusion criteria (N=101)	Publications (N=99)	Interventions (N=89)
1	Simmons, J., Motamedi, A., Ludvigsson, M., & Swahnberg, K. (2022). Testing an educational intervention to improve health care providers' preparedness to care for victims of elder abuse: a mixed method pilot study. *BMC medical education, 22*(1), 597.	Pilot study of an educational model aiming at improving health care providers' preparedness to care for older adults subjected to abuse
1	Mardomakdehi, A. F. S., Sum, S., & Faramarzi, M. (2020). Challenges for Detection of Elderly Abuse in Teaching Hospitals: A Interventional Study to Improve the Knowledge of Health Care Providers. *Shiraz E-Medical Journal, 21*(2).	EA detection empowerment workshop and screening protocol
1	Mariam, L. M., McClure, R., Robinson, J., & Yang, J. A. (2015). Eliciting change in at-risk elders (ECARE): Evaluation of an elder abuse intervention program. *Journal of elder abuse & neglect, 27*(1), 19-33.	Eliciting Change in At-Risk Elders (ECARE)
1	Martinez, J. M., Homeier, D. C., Fowler, C., & Wilber, K. H. (2023). Conceptualizing person-centered care in elder mistreatment intervention: Use of a well-being framework. *The Gerontologist, 63*(6), 973-982.	The Service Advocate Program
1	Matlaw, J. R., & Spence, D. M. (1994). The hospital elder assessment team: A protocol for suspected cases of elder abuse and neglect. *Journal of elder abuse & neglect, 6*(2), 23-38.	Elder Assessment Team (EAT)
1	Maxwell, C. D., Rodgers, K., & Pickering, C. E. (2022). Pragmatic Randomized Control Trial of a Coordinated Community Response: Increasing Access to Services for At-Risk Older Adults. *Journal of Forensic Nursing, 18*(2), 91-98.	Community complex care response team (C3RT)
1	McCauley, J., Jenckes, M. W., & McNutt, L.-A. (2003). ASSERT: the effectiveness of a continuing medical education video on knowledge and attitudes about interpersonal violence. *Academic medicine, 78*(5), 518-524.	Ask-Sympathize- Safety-Educate-Refer-Treat (ASSERT)
1	Mills, W. L., Roush, R. E., Moye, J., Kunik, M. E., Wilson, N. L., Taffet, G. E., & Naik, A. D. (2012). An educational program to assist clinicians in identifying elder investment fraud and financial exploitation. *Gerontology & Geriatrics Education, 33*(4), 351-363.	Elder investment fraud and financial exploitation (EIFFE) educational program
1	Mohd Mydin, F. H., Othman, S., Choo, W. Y., Hairi, N. N. M., Hairi, F. M., Syed Karim, S. N., . . . Mohd, R. (2021). Supporting family doctors to address elder abuse: a quasi-experimental study in Malaysia. *Journal of elder abuse & neglect, 33*(2), 151-167.	Supporting Family Doctors to Address Elder Abuse (SAFE) educational intervention

Number of interventions evaluated/described meeting inclusion criteria (N=101)	Publications (N=99)	Interventions (N=89)
1	Mohd Mydin, F. H., Wan Yuen, C., Othman, S., Mohd Hairi, N. N., Mohd Hairi, F., Ali, Z., & Abdul Aziz, S. (2022). Evaluating the effectiveness of I-NEED program: improving nurses’ detection and management of elder abuse and neglect—a 6-month prospective study. *Journal of interpersonal violence, 37*(1-2), NP719-NP741.	Improving Nurses’ dEtection and managEment of elDer abuse and neglect (I-NEED) intensive training program
1	Mosqueda, L., Burnight, K., Liao, S., & Kemp, B. (2004). Advancing the field of elder mistreatment: a new model for integration of social and medical services. *The Gerontologist, 44*(5), 703-708.	Vulnerable Adult Specialist Team (VAST)
1	Nakanishi, M., Nakashima, T., & Honda, T. (2010). Disparities in systems development for elder abuse prevention among municipalities in Japan: Implications for strategies to help municipalities develop community systems. *Social Science & Medicine, 71*(2), 400-404.	Japanese elder abuse prevention and caregiver support law
1	Nakanishi, M., Nakashima, T., Sakata, N., Tsuchiya, N., & Takizawa, K. (2013). Community-based system, reports, and substantiated cases of elder abuse: Disparities between municipalities and relating factors in Japan. *Journal of Aging & Social Policy, 25*(3), 234-247.	Long-Term Care Insurance (LCTI) program
1	Nouer, S. S., Meyer, L., Shen, Y., Hare, M. E., & Connor, P. D. (2020). Dental students’ perceived and actual knowledge of elder abuse: An online training curriculum. *Special Care in Dentistry, 40*(1), 106-112.	Dental students’ knowledge online training
1	Otaghi, M., Mousavimoghadam, S. R., Nikvand, M., & Khorshidi, A. (2022). The Effect of Appropriate Treatment Package for the Elderly on the Abuse of Rural Elderly Families in Eyvan City. *Asean Journal of Psychiatry, 23*(7).	Elder abuse training package on appropriate treatment
1	Oveisi, S., Jahed, S., & Olfati, F. (2018). Impact of counseling on the awareness of elderly women’s family about elder abuse according to the transtheoretical model. *Journal of Inflammatory Diseases, 22*(3), 32-39.	Counseling on the awareness of elderly women’s family
1	Oveisi, S., Stein, L., Olfati, F., & Jahed, S. (2021). Program development using intervention mapping in primary healthcare settings to address elder abuse: A randomized controlled pilot study. *Brain and Behavior, 11*(6), e02153.	"A culturally informed treatment, based on Intervention Mapping (IM), for primary healthcare settings"
1	Pandya, S. P. (2020). The effect of meditation in building resilience among older widows maltreated by children-in-laws. *Journal of Family Trauma, Child Custody & Child Development, 18*(1), 66-87.	Unnamed mediation program

Number of interventions evaluated/described meeting inclusion criteria (N=101)	Publications (N=99)	Interventions (N=89)
1	Park, D., & Ha, J. (2023). Education program promoting report of elder abuse by nursing students: a pilot study. *BMC geriatrics, 23*(1), 204.	Education program promoting intent to report elder abuse incidents among nursing students
1	Peisah, C., Bhatia, S., Macnab, J., & Brodaty, H. (2016). Knowledge translation regarding financial abuse and dementia for the banking sector: the development and testing of an education tool. *International journal of geriatric psychiatry, 31*(7), 702-707.	Adaptive learning tool for banking sector on financial abuse
1	Phillips, L. R. (2008). Abuse of aging caregivers: Test of a nursing intervention. *Advances in Nursing Science, 31*(2), 164-181.	Psychoeducative nursing intervention
1	Proehl, R. A. (2012). Protecting our elders: An interfaith coalition to address elder abuse. *Journal of Religion, Spirituality & Aging, 24*(3), 249-266.	Protecting Our Elders project
1	Rekawati, E., Hamid, A. Y. S., Sahar, J., Kamso, S., & Kusumawardani, L. H. (2020). The Effectiveness of the Cordial Older Family Nursing Model in Order to Improve the Quality of Family Care for Older Persons. *Indian Journal of Public Health Research & Development, 11*(2).	Cordial Older Family Nursing Model
1	Richardson, B., Kitchen, G., & Livingston, G. (2002). The effect of education on knowledge and management of elder abuse: a randomized controlled trial. *Age and ageing, 31*(5), 335-341.	Education on knowledge and management of elder abuse
1	Ries, N. M., & Mansfield, E. (2020). Action on elder abuse: A New South Wales pilot project on the role of legal and health practitioners in elder abuse screening, response and prevention. *University of New South Wales Law Journal, The, 43*(2), 738-761.	A pilot EA education and screening initiative
1	Rizzo, V. M., Burnes, D., & Chalfy, A. (2015). A systematic evaluation of a multidisciplinary social work–lawyer elder mistreatment intervention model. *Journal of elder abuse & neglect, 27*(1), 1-18.	Jewish Association Serving the Aging (JASA) Legal/Social Work Elder Abuse Prevention Program (LEAP) (JASA-LEAP)
1	Robson, C., Gutman, G., Marchbank, J., & Blair, K. (2018). Raising awareness and addressing elder abuse in the LGBT community: An intergenerational arts project. *Language and Literacy, 20*(3), 46-66.	Raising Awareness and Addressing Elder Abuse in the LGBT Community

Number of interventions evaluated/described meeting inclusion criteria (N=101)	Publications (N=99)	Interventions (N=89)
1	Rosen, T., Elman, A., Clark, S., Gogia, K., Stern, M. E., Mulcare, M. R., . . . Pearman, M. (2022). Vulnerable Elder Protection Team: Initial experience of an emergency d epartment-based interdisciplinary elder abuse program. *Journal of the American Geriatrics Society, 70*(11), 3260-3272.	Vulnerable elder protection team (VEPT)
1	Rosen, T., Mehta-Naik, N., Elman, A., Mulcare, M. R., Stern, M. E., Clark, S., . . . Lachs, M. (2018). Improving quality of care in hospitals for victims of elder mistreatment: Development of the vulnerable elder protection team. *The Joint Commission Journal on Quality and Patient Safety, 44*(3), 164-171.	Vulnerable Elder Protection Team (VEPT)
1	Ross, M. E. T., Bryan, J. L., Thomas, K. L., Asghar-Ali, A. A., & Pickens, S. L. (2020). Elder abuse education using standardized patient simulation in an undergraduate nursing program. *Journal of Nursing Education, 59*(6), 331-335.	Standardized Patient Simulation
1	Sadler, P., & Sorensen, G. (2000). Coordination and elder abuse: development of inter-agency protocols in New South Wales. *Australasian Journal on Ageing, 19*(3), 118-124.	Third training workshop for developing local inter-agency protocol
1	Sahar, J., Riasmini, N. M., & Nurviyandari, D. (2018). Reducing neglect and improving social support for older people following a self-help group in the poor urban community of Jakarta, Indonesia. *Enfermeria clinica, 28*, 66-69.	Self-help group using monitoring and intervention guidence
1	Seamon, J. P., Jones, J. S., Chun, E., & Krohmer, J. R. (1997). Identifying victims of elder abuse and neglect: a training video for prehospital personnel. *Prehospital and disaster medicine, 12*(4), 36-40.	Unnamed
1	Sirey, J. A., Berman, J., Salamone, A., DePasquale, A., Halkett, A., Raeifar, E., . . . Raue, P. J. (2015). Feasibility of integrating mental health screening and services into routine elder abuse practice to improve client outcomes. *Journal of elder abuse & neglect, 27*(3), 254-269.	Providing Options To Elderly Clients Together (PROTECT)
1	Sirey, J. A., Halkett, A., Chambers, S., Salamone, A., Bruce, M. L., Raue, P. J., & Berman, J. (2015). PROTECT: a pilot program to integrate mental health treatment into elder abuse services for older women. *Journal of elder abuse & neglect, 27*(4-5), 438-453.	Providing Options To Elderly Clients Together (PROTECT)

Number of interventions evaluated/described meeting inclusion criteria (N=101)	Publications (N=99)	Interventions (N=89)
1	Sirey, J. A., Solomonov, N., Guillod, A., Zanotti, P., Lee, J., Soliman, M., & Alexopoulos, G. S. (2021). PROTECT: a novel psychotherapy for late-life depression in elder abuse victims. *International psychogeriatrics, 33*(5), 521-525.	Providing Options To Elderly Clients Together (PROTECT)
1	Smith, D. E., Wright, M. T., Pham, T. H., & Ibrahim, J. E. (2022). Evaluation of an online course for prevention of unwanted sexual behaviour in residential aged care services—A pilot study. *International Journal of Older People Nursing, 17*(1), e12412.	Self guided e-learning educational course to prevent and manage unwanted sexual behaviour in residential aged care services
1	Sugita, J. A., & Garrett, M. D. (2012). Elder abuse and oral health care providers: an intervention to increase knowledge and self-perceived likelihood to report. *Journal of elder abuse & neglect, 24*(1), 50-64.	Elder Abuse Symposium for Oral Health Care Providers
1	Teresi, J. A., Ramirez, M., Ellis, J., Silver, S., Boratgis, G., Kong, J., . . . Lachs, M. S. (2013). A staff intervention targeting resident-to-resident elder mistreatment (R-REM) in long-term care increased staff knowledge, recognition and reporting: Results from a cluster randomized trial. *International Journal of Nursing Studies, 50*(5), 644-656.	Support, Evaluate, Act, Report, Care plan and Help (SEARCH)
1	Van Weert, J. C. (2004). *Multi-sensory stimulation in 24-hour dementia care.* Utrecht University,	Multi-Sensory Stimulation
1	Van Weert, J. C., Janssen, B. M., Van Dulmen, A. M., Spreeuwenberg, P. M., Bensing, J. M., & Ribbe, M. W. (2006). Nursing assistants' behaviour during morning care: effects of the implementation of snoezelen, integrated in 24-hour dementia care. *Journal of advanced nursing, 53*(6), 656-668.	Multi-Sensory Stimulation
1	Vyas, D., DelNero, T., Davenport, T. E., Musacchia, G., Nguyen, L., & Nguyen, A. (2021). Use of video vignettes and case discussions to educate health professions students about elder abuse. *Journal of Interprofessional Education & Practice, 24*, 100434.	Interprofessional education (IPE) program
1	West, A., Cawley, C., Crow, E., Stoner, A. M., Fadel, N. M., Ford-Scales, K., & Cheng, N. (2021). The impact of an educational program on medical students' knowledge and awareness of elder abuse. *Journal of medical education and curricular development, 8*, 23821205211016487.	Student Training on Preventing Domestic Violence (STOP-DV)

Appendix 4. Publication Year of the Promising Interventions (101 intervention evaluations or descriptions)

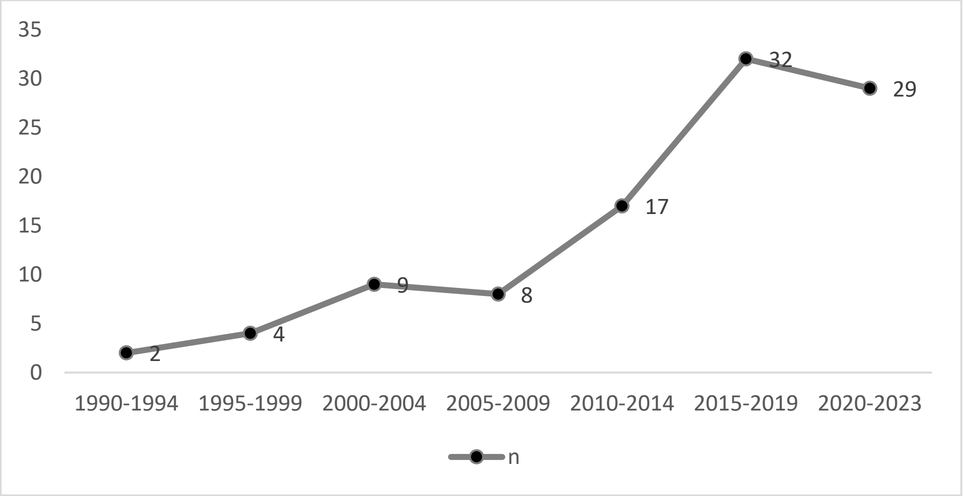

Appendix 5. Number of Promising Interventions to Address Abuse of Older People Per Country (n = 101).

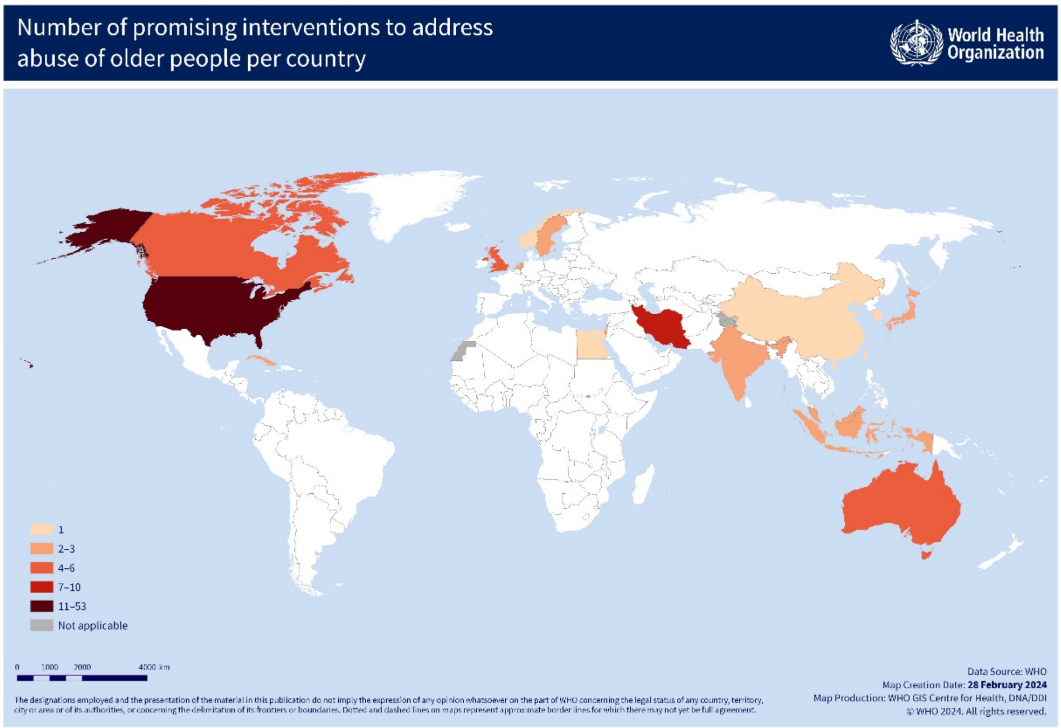

Supplement: Campo-Tena et al Appendix [file NIHMS2042655-supplement-Campo-Tena_et_al_Appendix.pdf]
